# Maternal Depressive Symptoms and Risk for Childhood Depression: Role of Executive Functions

**DOI:** 10.1016/j.jaac.2024.08.503

**Published:** 2024-10-16

**Authors:** Meredith X. Han, Ranjani Nadarajan, Nixi Wang, Michelle Z.L. Kee, Shuping Lim, Yashna K. Sagar, Benjamin Chow, Ai Peng Tan, Bobby K. Cheon, Yuen-Siang Ang, Juan H. Zhou, Helen Y. Chen, Yap Seng Chong, Peter D. Gluckman, Michael J. Meaney, Evelyn C. Law

**Affiliations:** Yong Loo Lin School of Medicine, National University of Singapore, Singapore, Singapore, Institute of Psychiatry, Psychology and Neuroscience, King’s College London, London, United Kingdom; Douglas Hospital Research Centre, McGill University, Montreal, Quebec, Canada; Yong Loo Lin School of Medicine, National University of Singapore, Singapore, Singapore; Institute for Human Development and Potential, Agency for Science, Technology and Research (A*STAR), Singapore, Singapore; Yong Loo Lin School of Medicine, National University of Singapore, Singapore, Singapore; Yong Loo Lin School of Medicine, National University of Singapore, Singapore, Singapore; Institute for Human Development and Potential, Agency for Science, Technology and Research (A*STAR), Singapore, Singapore; Institute for Human Development and Potential, Agency for Science, Technology and Research (A*STAR), Singapore, Singapore, National University Hospital, Singapore, Singapore; Eunice Kennedy Shriver National Institute of Child Health and Human Development, National Institutes of Health, Bethesda, Maryland; Institute for High Performance Computing, A*STAR, Singapore, Singapore; Yong Loo Lin School of Medicine, National University of Singapore, Singapore, Singapore; KK Women’s and Children’s Hospital and Duke-NUS Medical School, Singapore, Singapore; Yong Loo Lin School of Medicine, National University of Singapore, Singapore, Singapore, National University Hospital, Singapore, Singapore; Institute for Human Development and Potential, Agency for Science, Technology and Research (A*STAR), Singapore, Singapore, Liggins Institute, University of Auckland, Auckland, New Zealand; Douglas Hospital Research Centre, McGill University, Montreal, Quebec, Canada, Institute for Human Development and Potential, Agency for Science, Technology and Research (A*STAR), Singapore, Singapore; Yong Loo Lin School of Medicine, National University of Singapore, Singapore, Singapore, Institute for Human Development and Potential, Agency for Science, Technology and Research (A*STAR), Singapore, Singapore, National University Hospital, Singapore, Singapore

**Keywords:** child development, depression, executive function, intergenerational relations, mediation analysis

## Abstract

**Objective:**

Offspring of mothers with depression are at increased risk for executive function (EF) deficits and later depressive symptoms, but limited studies have examined EF as an intermediary pathway. This study examined the role of EF in mediating the association between maternal and child depressive symptoms.

**Method:**

Data were from a longitudinal birth cohort comprising 739 participants followed from the antenatal period for 12 years. Mothers completed the Edinburgh Perinatal Depression Scale at 26 to 28 weeks’ gestation and at 3 and 24 months postpartum. At ages 8.5 to 10 years, children self-reported using the Children’s Depression Inventory, Second Edition. Task-based and parent-reported EF measures were collected at 4 time points between 3.5 and 8.5 years. Latent growth curve models examined antenatal depressive symptoms and their trajectory in contributing to cold (ie, cognitive) and hot (ie, affective) EFs. The extent to which EF mediated this association was then assessed.

**Results:**

Maternal depressive symptoms did not directly predict depressive symptoms in late childhood. Antenatal depressive symptoms predicted lower cold EF (*β* = −.13, 95% CI [−0.25, −0.004]) and hot EF (*β* = −.26, 95% CI [−0.38, −0.15]). Deficits in cold EF (*β* = −.26, 95% CI [−0.41, −0.11]) acted as an intermediary path to depressive symptoms, whereas hot EF mediated the association between maternal and child depressive symptoms, forming an indirect path that accounted for 37.5% of the association.

**Conclusion:**

Deficits in hot EF may be a pathway in explaining the intergenerational transmission of depression. This finding suggests fostering EF skills as a potential strategy for at-risk children.

**Clinical trial registration information:**

Growing Up in Singapore Towards Healthy Outcomes (GUSTO); https://clinicaltrials.gov/study/NCT01174875?cond=NCT01174875

**Plain language summary:**

This study using data from the Growing Up in Singapore Towards Healthy Outcomes cohort (n=739) examines the role of cognitive and affective executive functions (EF) in mediating the association between maternal and child depressive symptoms. Results show that “hot” EF (regulating emotions and motivation) accounted for 37.5% of the association between maternal prenatal depression and childhood depressive symptoms. Findings suggest that fostering hot EF skills may be a preventative strategy for children from families at risk of intergenerational transmission of depression.

**Diversity & Inclusion Statement:**

We worked to ensure that the study questionnaires were prepared in an inclusive way. We worked to ensure sex and gender balance in the recruitment of human participants. We worked to ensure race, ethnic, and/or other types of diversity in the recruitment of human participants. One or more of the authors of this paper self-identifies as a member of one or more historically underrepresented racial and/or ethnic groups in science. We actively worked to promote sex and gender balance in our author group. We actively worked to promote inclusion of historically underrepresented racial and/or ethnic groups in science in our author group. While citing references scientifically relevant for this work, we also actively worked to promote sex and gender balance in our reference list. While citing references scientifically relevant for this work, we also actively worked to promote inclusion of historically underrepresented racial and/or ethnic groups in science in our reference list. The author list of this paper includes contributors from the location and/or community where the research was conducted who participated in the data collection, design, analysis, and/or interpretation of the work. One or more of the authors of this paper self-identifies as a member of one or more historically underrepresented sexual and/or gender groups in science. One or more of the authors of this paper self-identifies as living with a disability.

Perinatal maternal depression is an established risk factor for socioemotional problems in the offspring.^1,2^ Despite extensive epidemiological research linking maternal mental health with offspring mood outcomes, the underlying pathways remain to be clarified. Competency models of child psychopathology suggest that competence in one domain acts as a scaffold to foster competencies in other emerging domains.^3,4^ Conversely, the inability to achieve age-appropriate goals may increase the risk for mental health problems.^4^ Executive function (EF) is a key competency as it allows children to achieve success in academic pursuits and social interactions.^5–7^ Studies suggest that childhood EF may have a long-lasting influence, as it appears to decrease the risk for mental health disorders in adolescence.^8,9^ Therefore, one possible pathway contributing to the intergenerational transmission of risk for depression may involve EF.

Accumulating evidence supports the division of EF into its specialized functions.^10^ Cold EF refers to higher-order cognitive skills that are analytical and consists of the subdomains of working memory, inhibitory control, and cognitive flexibility.^7,10^ In contrast, hot EF refers to skills that are elicited within emotionally-laden contexts and/or risk-involved contexts (eg, emotional control).^10^ Evidence for the division of EF is supported by behavioral and neuroimaging studies.^11–13^ Functional neuroimaging studies show increased activity in the medial orbitofrontal cortex during risky decision making (ie, low probabilities of rewards, high probability of losses), whereas increased activity is seen in the dorsolateral prefrontal cortex during tasks requiring cognitive control and decision making.^12,14^ Similarly, patients with medial orbital-prefrontal cortex lesions show impairments in tasks involving risky decision making but are unimpaired on tasks measuring cognitive flexibility.^13^ Of note, hot and cold EFs do not perform independently of each other. Emotional regulation deficits can impair working memory and vice versa.^13^ As such, deficits in both cold and hot EFs confer risk via separate, but interacting, pathways. Nonetheless, given the diversity of functions, the distinction of EF offers a practical framework for examining cognitive processes involved in psychopathology.

Cold and hot EF deficits are found in individuals with clinical depression.^15,16^ A review of neuroimaging studies conducted in children with clinical depression reveals structural and functional alterations to dorsolateral prefrontal cortex, anterior cingulate, and caudate that are involved in attention processes as well as ventromedial prefrontal cortex–limbic circuitries involved in emotion regulation and motivation.^16^ Interestingly, functional magnetic resonance imaging studies demonstrate that the fronto-striatal-thalamic circuitries are impaired in these children only when there is a motivation component in the sustained attention task.^17,18^ These findings also translate into behavioral findings.^19–21^ For example, individuals with depression demonstrate greater anger reactivity and less persistence on a frustration-eliciting task.^21^ Similarly, when assessing emotion regulation in school-age children via parent- and child-reported questionnaires, results reveal that higher emotional control is associated with lower depressive symptoms.^22,23^

Studies also show an association between individual subdomains of cold EF and internalizing problems. Children with difficulties in working memory, cognitive flexibility, and inhibitory control were found to have an increased incidence of internalizing disorders.^20,24,25^ In particular, using laboratory-based tasks, school-age children with more internalizing symptoms made more errors on the inhibitory Go/No-go and spatial working memory tasks.^25^ Likewise, interventions such as the Family Check-Up intervention, which indirectly improve EF via positive parenting behaviors during childhood, were associated with reduced emotional difficulties in adolescence.^26^ Therefore, EF competencies may help to foster adaptive behavior, enabling children to overcome developmental challenges across home, educational, and social settings.^3^

There are 3 main research gaps in examining the transmission of risk for depression from mothers to children. First, few studies have explored the specific process through which maternal depression may confer risk for offspring depressive symptoms. Specifically, the role of hot and cold EFs as plausible intermediary paths has not been explored in a single model. Thus far, 1 longitudinal birth cohort study found that cold EF as a latent variable, consisting of working memory, cognitive flexibility, inhibitory control, and planning at age 3, mediated the relation between maternal postnatal depressive symptoms and child externalizing and internalizing problems at age 6.^27^ Additionally, Silk *et al.*^28^ demonstrated that emotion regulation, assessed using a delay gratification task, moderated the influence of maternal history of depression on child internalizing problems. However, both studies were limited by a short assessment period and the examination of 1 EF domain. Given the distinct functions of hot and cold EFs, a more comprehensive understanding of their individual and joint contributions to child depressive symptoms is warranted. Second, prior studies examining the role of EF in childhood depression were limited by the use of parent-reported questionnaires to assess child EF or relied on few measures of EF.^28–30^ Lastly, mixed findings are reported on the extent to which antenatal or postnatal maternal depressive symptoms influence the development of child EF and depressive symptoms.^30–32^ One study found that chronic exposure to maternal depression within the first 2 years after birth contribute to EF difficulties at age 9.^30^ However, analyses using larger samples, notably those of the Avon Longitudinal Study of Parents and Children (ALSPAC), demonstrated that antenatal depressive symptoms were more strongly associated with the risk for offspring depression than postnatal symptoms.^1^

This study aimed to address these gaps by incorporating comprehensive EF assessment that consisted of validated parental reports and laboratory-based tasks measuring multiple domains of hot and cold EF from ages 3.5 to 8.5 years. Our analysis focused on maternal depressive symptoms assessed over the antenatal period into the first 2 postnatal years, which included 3 time points at midgestation, 3 months postpartum, and 24 months postpartum. This approach allowed us to explore the influence of antenatal depressive symptoms and the trajectory from antenatal to postnatal symptoms. We also sought to understand whether a 2-factor structure of cold EF fit our data more than a 3-factor structure of EF (namely, inhibitory control, working memory, and cognitive control) commonly seen in adults.^7^ Based on theory and previous literature, we hypothesized that a 2-factor structure of cold EF, consisting of cognitive control and working memory, fit our data more than a 3-factor structure.^33,34^ We also hypothesized that both cold and hot EFs would mediate the association between maternal depressive symptoms during pregnancy and offspring symptoms of depression.

## METHOD

### Study Design and Participants

The Growing Up in Singapore Towards Healthy Outcomes (GUSTO) cohort is a prospective longitudinal birth study of the developmental origins of health and human capital. Women 18 to 40 years of age across all socioeconomic backgrounds were recruited during their first trimester of pregnancy from the 2 main public birthing hospitals in Singapore between June 2009 and December 2010.^35^ Both parents belonged to 1 of the 3 major ethnicities in Singapore (ie, Chinese, Indian, or Malay). Mother–child dyads were followed throughout pregnancy and beyond. We excluded children who were born via in vitro fertilization, with a history of prematurity before 32 weeks’ gestation, and with genetic and neurological conditions. The study was approved by the Institutional Review Board of both hospitals. All mothers signed an informed consent before enrollment in the cohort, and all children assented to the study at 7 years of age. Children who exhibited clinical levels of depression and/or were at risk of self-harm on mental health–related measures were referred to health care services and received routine care and interventions. Families were offered SGD 50.00 (USD $37.40) for 3- to 5-hour testing session in the laboratory at ages 8.5 to 9 years.

### Procedures

Antenatal maternal depressive symptoms collected at 26 to 28 weeks’ gestation, 3 months postpartum, and 24 months postpartum were analyzed as continuous variables. EF assessments were carried out during 4 visits at the research center when children were ages 3.5, 4.5, 7, and 8.5 years. Details of each assessment are found in Table S1 (available online). As English is the main language of educational instruction in Singapore, we administered all EF tasks in English. Trained research assistants assessed children in the laboratory using computer-based tasks for measures of cold EF. Reliable coding methods from video recordings were used for hot EF. The intraclass correlation coefficients on these video recordings ranged from 0.64 to 0.97.^29^ Child depressive symptoms were collected through self-reported questionnaires in 2 waves: at age 8.5 to 9 years (wave 1) and at age 10 years (wave 2). We prioritized the first measure for participants who completed the questionnaire at both 8.5 and 10 years.

### Maternal Depressive Symptoms

Self-reported depressive symptoms were assessed with the Edinburgh Perinatal Depression Scale (EPDS) at 26 to 28 weeks’ gestation, 3 months postpartum, and 24 months postpartum. The EPDS is a validated self-report instrument that contains 10 items of common depressive symptoms over the past week. The EPDS is validated for antenatal and postnatal screening for depression.^36^ Scores range from 0 to 30 with higher scores indicating more symptoms.

#### Cold EF.

Cold EF comprised 2 established subdomains: working memory (ie, the ability to hold in mind and manipulate representations) and cognitive control, which consisted of inhibitory control (ie, the ability to inhibit prepotent responses) and cognitive flexibility (ie, the ability to adapt behavior according to environmental demands). This differentiation is consistent with the structure of cold EF in childhood.^33,34^ Previous studies examining cold EF in relation to internalizing disorders have included similar tasks and questionnaires.^20,24,25^

#### Working Memory.

Spatial working memory from the Cambridge Neuropsychological Test Automated Battery (CANTAB) was assessed at age 4.5 years (n = 455). The child was tasked with searching for blue tokens behind 6 boxes on a computer screen. The goal was to find all the tokens without reopening a box. The mean number of times a child revisited an already opened box with a token (ie, between error) and without the token (ie, within error) were used in the analysis. The Developmental Neuropsychological Assessment, Second Edition (NEPSY-II) was used at age 8.5 years (n = 436).^37^ The word interference subtest in NEPSY-II measured the ability to hold in memory verbal information with interference. Higher recall scaled scores indicate better competency in word-level working memory. The working memory subscale as a composite score from the Behavior Rating Inventory of Executive Function, Second Edition, Parent and Teacher Rating Forms (BRIEF-2) (n = 620) assessed the ability to retain information when completing tasks at ages 7 and 8.5 years.^38^ A composite score from parent and teacher BRIEF-2 reports was created. A higher T score indicates greater working memory deficits. BRIEF-2 is a valid tool to assess EF difficulties in Asian children with a Cronbach β of .66 to .89.^39^

#### Cognitive Control Latent Variable.

Cognitive control consisted of measures of cognitive flexibility and inhibitory control. The stop-reaction time task (n = 394) was used at 4.5 years to measure inhibitory control.^40^ Cognitive flexibility was assessed using a modified version of the Dimensional Card Change Sort (DCCS) at 4.5 years (n = 342).^41^ The inhibitory subscale from BRIEF-2 was used to measure the ability to resist impulses at 7 years. A higher T score indicates greater inhibitory control deficits. Cognitive flexibility was assessed at 8.5 years using the NEPSY-II inhibition subtest. Higher scores demonstrate better competency in cognitive flexibility.

#### Hot EF.

Hot EF comprised the proposed subdomains of emotional control (ie, the ability to regulate emotions in stress-inducing situations) and motivational control (ie, the ability to persevere through challenges to reach goals without external incentives).^10,20,21,23^ The emotional control subscale from the Behavior Rating Inventory of Executive Function, Preschool version, Parent Rating Form (BRIEF-P) (n = 429)^42^ was used to assess the ability to modulate or control emotional states at age 4.5 years. The emotion regulation subscale from parent- and teacher-reported BRIEF-2 was used to measure the ability to regulate emotional responses and adjust to environmental changes at ages 7 and 8.5 years. In both questionnaires, higher T scores indicate emotional control and regulation deficits. In the impossible tangram task (n = 458), children were given 3 sets of tangrams and were asked to replicate them using wooden shapes. However, unbeknown to the children, the third puzzle was impossible to solve. Children were rated on their frequency of anger and frustration behaviors on a scale of 1 to 5. A higher score indicates poorer emotional regulation.

The latent variable motivational control consisted of delayed gratification, the ability to resist immediate gratifying rewards, and persistence, the ability to sustain effort to achieve a desired goal. Delayed gratification was measured by snack delay and sticker delay tasks at 3.5 years (n = 415).^43^ Motivational control was assessed using the Laboratory Temperament Assessment Battery (Lab-TAB) transparent box task at 3.5 years (n = 370) and the impossible tangram task (n = 458).^44^ In the Lab-TAB task, a desirable toy was placed in a transparent box. The experimenter left the child with an incorrect set of keys and instructed them to open the box. The time expended in opening the transparent box was assessed. Higher score in the time expended indicates better motivational control. Similarly, in the impossible tangram task, a higher score in persistence demonstrates greater motivational control.

### Child Depressive Symptoms

Depressive symptoms were obtained from children at ages 8.5 and 10 years using the 28-item Child Depression Inventory, Second Edition (CDI-2) (n = 739).^45^ CDI-2 is a self-reported questionnaire that consists of 4 subscales—negative mood/physical symptoms, low self-esteem, ineffectiveness, and interpersonal problems—which together make up a composite T score. This T score was produced after accounting for sex and age of the child, with a mean (SD) score of 50 (10.) A T score above 65 on the CDI-2 indicates elevated depressive symptoms. CDI-2 is a valid screening tool for depressive symptoms in children in Singapore, with a Cronbach β of 0.67 to 0.91.^46^

### Statistical Analysis

Descriptive statistics using χ^2^ and independent t tests were used to compare maternal and child demographic variables between the entire GUSTO cohort (N = 1,195) and a subsample with data on child depressive symptoms (n = 739). Multivariable linear regression examined the relation between maternal perinatal and child depressive symptoms and between individual EF measures at ages 3.5 to 8.5 years and child depressive symptoms. We controlled for monthly household income reported by parents during pregnancy, maternal depressive symptoms at 26 weeks’ gestation, age at CDI-2 data collection, sex, and ethnicity in 3 categories (ie, Chinese, Indian, Malay), all of which have been associated with childhood depressive symptoms.^47^ Given that externalizing problems had been shown to be comorbid with depressive symptoms, we included externalizing problems (measured using the externalizing subscale from the Child Behavior Checklist [CBCL])^48^ as an additional covariate in the linear regression.

We first used a structural equation modeling approach to examine how all the indicators loaded onto 4 EF latent variables (ie, working memory, cognitive, emotional, and motivational control), then we modeled their association with CDI-2 total score. Latent growth curve model was performed to test whether initial antenatal depression (intercept) contributed to EF differently than maternal depression over time (slope from 26 weeks’ gestation to 3 and 24 months postpartum). We then examined the separate contributions of cold EF (ie, working memory and cognitive control) and hot EF (ie, motivational and emotional control) in explaining the path from maternal to child depressive symptoms. Considering the longitudinal nature of our measures (ie, EF measures were collected at different ages), we modeled correlations among the residual terms of measures collected within the same time point. Mediation analyses with maximum likelihood robust estimation were then conducted with hot and cold EF constructs in the same model. In our final models, the individual contributions of working memory, cognitive control, emotional control, and motivational control to child symptoms of depression were examined.

Appropriate model fit was assessed using the following indices: root mean square error of approximation <0.05, standardized root mean square residual <0.07, comparative fit index >0.90, Akaike information criteria , and the model χ^2^ test.^49^ Data were analyzed using STATA version 15.1 (StataCorp LLC, College Station, Texas) and Mplus version 8.6.^50^

## RESULTS

At ages 8.5 and/or 10 years, 739 children provided data on self-reported depressive symptoms on CDI-2 (Figure S1, available online). The mean (SD) age of assessment was 9.48 (0.65) years. Table 1 shows the characteristics of the GUSTO cohort and children in our sample with CDI-2 data. No differences were found (all *p* > .05). The study sample consisted of 58% Chinese, 15% Indian, and 27% Malay families. In our sample, 9.5% of mothers reported clinical levels of depressive symptoms (EPDS ≥15),^51^ and 26.5% of children reported elevated depressive symptoms (T score >65). More than 15% of families reported a household income of less than SGD 2,000 per month (approximately USD $1,495). We also found that mothers and children belonging to Indian and Malay ethnicities reported higher levels of depressive symptoms compared with Chinese participants (Table S2, available online).

### Perinatal Maternal and Child Depressive Symptoms

We examined the relation between perinatal depressive symptoms at 26 to 28 weeks’ gestation, 3 months postpartum, and 24 months postpartum in linear regressions (Table S3, available online). Only antenatal maternal symptoms of depression were significantly correlated with CDI total score in the child (*β* = .22, 95% CI [0.02, 0.42], *p* = .035).

### EF Measures Predicting Child Depressive Symptoms

Table 2 shows the results in the multivariable linear regression models associating EF measures with child CDI-2 total score. After controlling for multiple comparisons and sociodemographic factors, working memory, cognitive control, and emotional control measures contributed to childhood depressive symptoms with small to moderate effect sizes (η^2^_P_ = 0.01-0.03). Ethnicity, when adjusted in the analyses, did not change the association between antenatal depressive symptoms, EF, and child depression.

### Latent EF Constructs

A correlation matrix of EF measures demonstrated moderate correlations for items measured using the same task and lower correlations for items measured within the same time point (Table S4, available online). Hot EF latent construct included both parent-reported and laboratory-based tasks, as this demonstrated the best model fit. In contrast, given the availability of objective cold EF measures, we excluded parent-reported measures in our cold latent construct to reduce the influence of shared-reporter bias. We found that the 3-factor structure of cold EF (ie, working memory, inhibitory control, and cognitive flexibility) did not provide an adequate fit to our data, particularly the construct of inhibitory control (Figure S2, available online).^7^ We then proceeded with an established 2-factor cold EF model by combining inhibitory control with cognitive flexibility to form cognitive control (Figure S3, available online).^33,34^ Loadings of all items to their respective latent variable were satisfactory, and the fit indices were good to exceptional, suggesting that these items contributed to the same EF construct (Table S5, available online).

### EF Individual Latent Models

The independent contributions of cold and hot EF subdomains to child depressive symptoms were examined. Model paths demonstrated that lower working memory (*β* = −.24, 95% CI [−0.40, −0.09], *p* = .002) and cognitive control competency (*β* = −.32, 95% CI [−0.51, −0.14], *p* = .001) and increased emotional control deficits (β = .14, 95% CI [0.03, 0.26], *p* = .012) were associated with increased child depressive symptoms (Figure S3, Figure S4, and Table S6, available online). No association was found between motivational control and child depressive symptoms.

### Latent Growth Curve Model

We tested the differential contributions of initial antenatal maternal depressive symptoms and the trajectory of depressive symptoms from antenatal to postpartum on child cold EF and hot EF (Figure S5, available online). Both antenatal depressive symptoms (*β* = −.45, 95% CI [−0.59, −0.32], *p* < .001) and the trajectory of maternal depressive symptoms into the postpartum period (*β* = −.26, 95% CI [−0.49, −0.02], *p* = .035) were negatively associated with hot EF competency. No association was found for the initial status and linear growth of maternal depressive symptoms and cold EF.

### Relations Between Maternal Depressive Symptoms, EFs, and Depressive Symptoms

**FIGURE 1:**
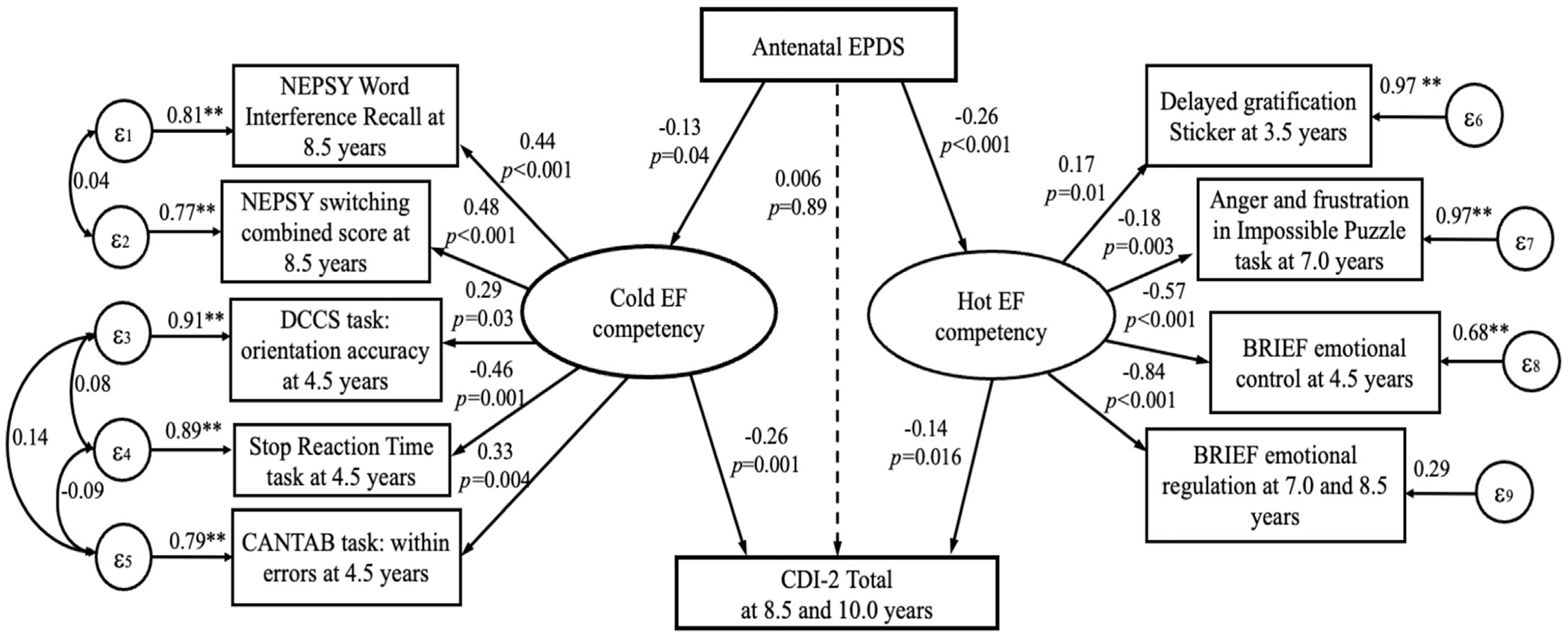
Structural Equation Model Examining Executive Functions in the Path From Maternal to Child Depressive Symptoms^a^ **Note:** Numbers are standardized β values (p value). ε indicates measurement errors of the endogenous variables in the structural equation model. BRIEF= Behavior Rating Inventory of Executive Function, Parent and Teacher Rating Forms (parent-reported at 4.5 years, teacher- and parent-reported at 7.0 and 8.5 years); CANTAB = Cambridge Neuropsychological Test Automated Battery; CDI-2 = Child Depression Inventory, Second Edition; DCCS= Dimensional Card Change Sort; EF = executive function; EPDS = Edinburgh Perinatal Depression Scale; NEPSY= Developmental Neuropsychological Assessment. ^a^ Correlation terms are added to measures collected within the same wave: *p < .05, **p < .001.

Following the latent growth curve model results, we focused our model on the antenatal period as the exposure (Figure 1). Antenatal maternal depressive symptoms predicted poorer cold EF (*β* = −.13, 95% CI [−0.25, −0.004], *p* = .042) and hot EF (*β* = −.26, 95% CI [−0.38, −0.15], *p* < .001). Poorer cold EF (*β* = −.26, 95% CI [−0.41, −0.11], *p* = .001) and hot EF (*β* = −.14, 95% CI [−0.25, −0.03], *p* = .02) competency in turn contributed to greater child depressive symptoms. There was no direct path from antenatal maternal depressive symptoms to child depressive symptoms in this final model. The mediation analysis showed that only hot EF provided an indirect path (*β* = .03, 95% CI [0.004, 0.07], *p* = .028). This path accounted for 37.5% of the total effects (Table S6, available online). Including maternal reports for hot EF could inflate parent–offspring correlation via shared method variance. Therefore, we also conducted supplementary analyses using only a laboratory-based hot EF task (Supplement 1 and Table S7, available online).

### EF Subdomains on Child Depressive Symptoms

**FIGURE 2:**
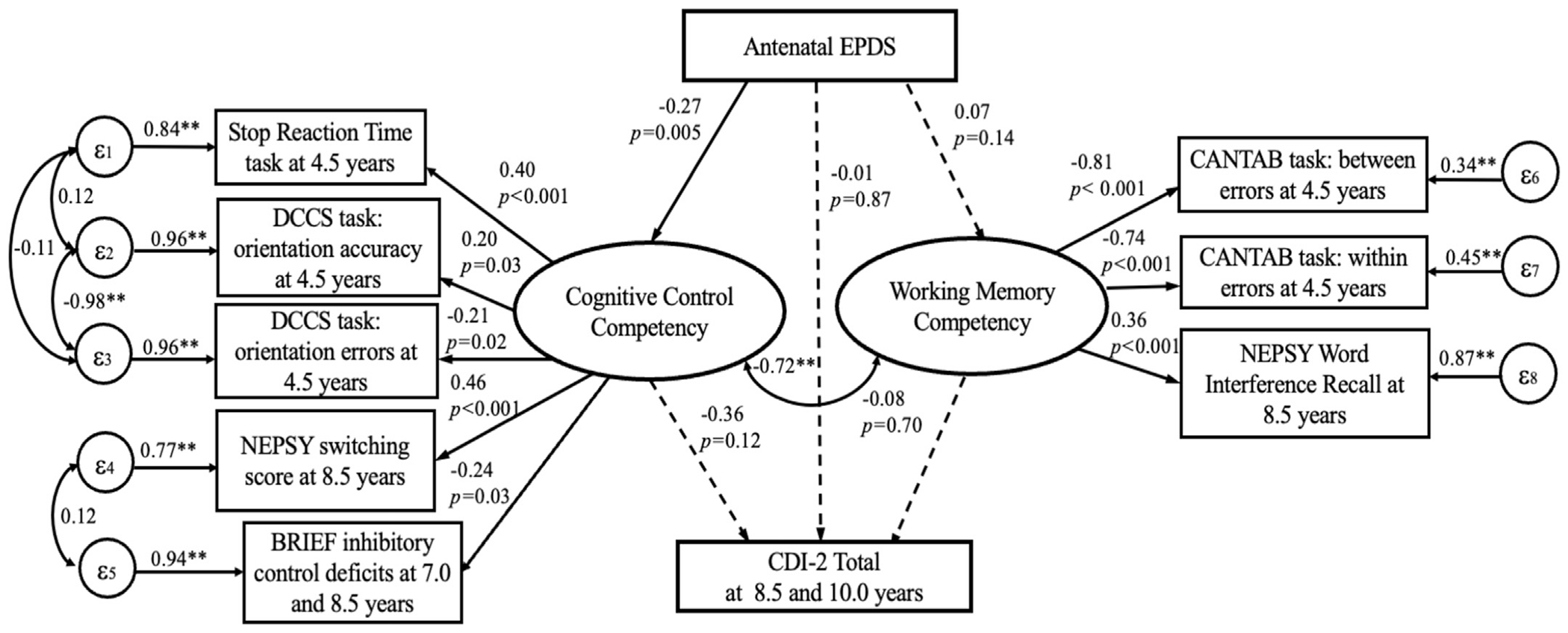
Structural Equation Model Examining the Contribution of Cognitive Control and Working Memory on Child Depressive Symptoms **Note:** Numbers are standardized β value (p value). ε indicates measurement errors of the endogenous variables in the structural equation model. BRIEF= Behavior Rating Inventory of Executive Function, Parent and Teacher Rating Forms (parent-reported at 4.5 years, teacher- and parent-reported at 7.0 and 8.5 years); CANTAB= Cambridge Neuropsychological Test Automated Battery; CDI-2 = Child Depression Inventory, Second Edition; DCCS= Dimensional Card Change Sort; EPDS = Edinburgh Perinatal Depression Scale; NEPSY= Developmental Neuropsychological Assessment. * p < .05, **p < .001.

**FIGURE 3:**
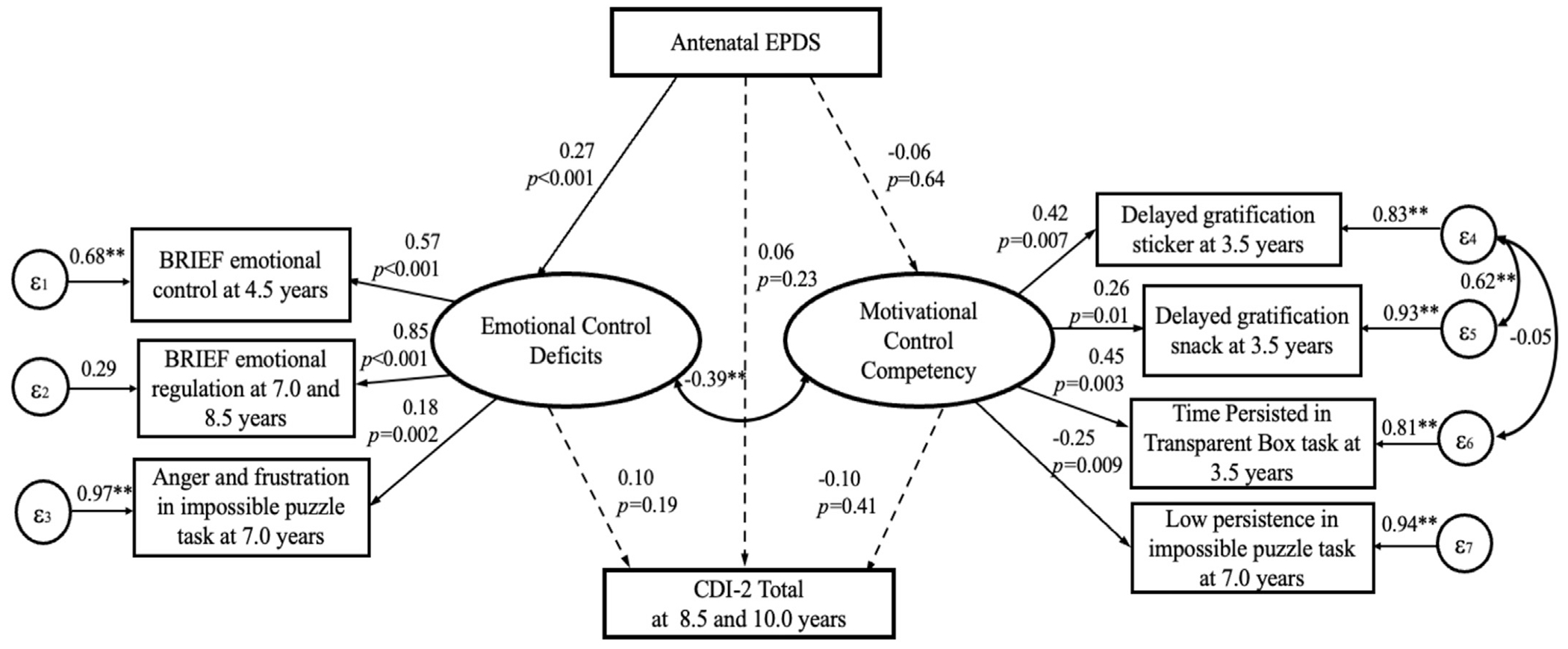
Structural Equation Model Examining the Contribution of Emotional Control and Motivational Control on Child Depressive Symptoms **Note:** Numbers are standardized β value (p value). ε indicates measurement errors of the endogenous variables in the structural equation model. BRIEF = Behavioral Rating Inventory of Executive Functions (parent-reported at 4.5 years, teacher- and parent- reported at 7.0 and 8.5 years); CDI-2 = Child Depression Inventory, Second Edition; EPDS = Edinburgh Perinatal Depression Scale. *p < .05, **p < .001.

We sought to examine which subdomain of EF was most implicated in the path from maternal to offspring depressive symptoms. Greater maternal antenatal depressive symptoms contributed to lower cognitive control (*β* = −.27, 95% CI [−0.45, −0.08], *p* = .005) and higher emotional control deficits (*β* = .27, 95% CI [0.15, 0.36], *p* < .001) (Figures 2 and 3). EF subdomains on their own were not associated with child depressive symptoms. Mediation analyses did not reveal any indirect paths provided by the individual subdomains (Table S8, available online).

## DISCUSSION

Maternal depression is a well-established risk factor for cognitive and emotional deficits in the offspring.^1,27^ We used parent-reported questionnaires and laboratory-based tasks to examine hot and cold EF as plausible mediators in the association between maternal depressive symptoms, from pregnancy to 2 years postnatal, and offspring symptoms of depression in mid-childhood. Our findings confirmed that antenatal maternal depressive symptoms predicted the variance in offspring EF deficits. Hot EF mediated this maternal–child association, whereas cold EF acted as an intermediary variable.

This association between antenatal depression and child EF deficits was consistent with previous neuroimaging studies reporting associations between exposure to antenatal maternal depressive symptoms and alterations in the prefrontal cortex in the offspring shortly after birth.^1,51,52^ Specifically, antenatal maternal depressive symptoms were associated with white microstructure alterations within the dorsolateral prefrontal cortex (a region critical for cold EF development) in 1-month-old offspring.^52^ Another study from our group conducted in 6-month-old infants revealed an association between antenatal depressive symptoms and infant neural connectivity between prefrontal regions and the right amygdala, which were areas implicated in emotion regulation.^53^ Alterations in connectivity were also shown to persist into preschool years.^54^ The relevance of these findings was underscored by other studies demonstrating reduced cortical thickness and altered neural connectivity as risk factors for clinical depression.^55,56^ These prior studies, taken together with behavioral data from this study, suggested an association between antenatal maternal depressive states and brain structures important for cold and hot EFs. However, we note that the trajectory of maternal depression into the postpartum period was associated with hot EF. This finding supported previous research on the association between chronic exposure of maternal depression on child psychopathology, reflecting the additional importance of early-life caregiving and social learning mechanisms in the development of emotional and motivational control.^30,57,58^

This study shows support that hot EF mediates the association between maternal and child depressive symptoms, which is consistent with findings from previous studies and the competency model of child psychopathology. The competency-based model of child depression posits that having competency in daily living skills, including EF and academic skills, is associated with positive self-concept, which in turn is protective of the development of childhood depression.^3,4,20,21,23^ In particular, emotional control is strongly associated with greater peer acceptance, more positive friendship quality, and higher academic achievement.^6,59^ Neuroimaging studies show that alterations in the medial prefrontal and temporal lobe regions implicated in hot EF precede the onset of depressive symptoms in adolescence.^60^ The ability to recruit prefrontal brain regions to regulate emotions is also associated with lower emotional symptoms in children following exposure to maltreatment.^61^ The underlying mechanism of how emotion regulation reduces depressive symptoms remains to be established. Recent studies suggest that emotion regulation may enable effective cognitive reappraisal of negative events, reducing negative affect.^20,62^ Deficits in emotion regulation may also be due to a lack of physiological adaptation to situational demands as seen by reduced respiratory sinus arrhythmias in children during a frustration-inducing task.^20^ Thus, our findings suggest a potential mediating pathway via EF development of the offspring that explains the intergenerational transmission of depressive symptoms. However, the mediation should be interpreted with caution as using the same reporter in certain instances across exposure and outcome in our primary analyses may have inflated associations via shared method variance. This is a possibility as our sensitivity analyses using a smaller sample of participants with laboratory-based tasks do not show the same mediation. However, we have reduced this possibility in our primary analyses by incorporating teacher reports and objective laboratory-based tasks. This also maximizes the value of a multi-informant approach by capturing observations across home and school contexts.^63^ An alternative explanation for the difference in results is that the laboratory-based hot EF measures may not be capturing the same underlying construct as questionnaire-based measures. Parent reports and laboratory tasks capture emotional and motivational control behaviors from a more ecological vs objective perspective, with different biases associated with each method.^63,64^ Hence, variation in research methodology and measures used in each study must be carefully considered when interpreting results.

Only hot EF, and not cold EF, mediated the relation between maternal and child depressive symptoms. Cold EF, however, acted as an intermediary variable whereby it demonstrated separate associations with the exposure and outcome without forming an indirect mediation path. Therefore, it is likely that cold EF increases the risk for child depression via other more proximal environmental factors. For example, in a longitudinal analysis, peer difficulties mediated the relation between cold EF, consisting of subdomains of inhibitory control, planning and working memory, and internalizing problems from ages 8 to 12.^65^ This suggests that cold EF may have cascading effects on competencies that are more closely related to emotional functioning.^3^

When examining the individual subdomains of cold EF and hot EF, working memory and motivational control were not found to be implicated in the continuity of depressive symptoms from mothers to offspring. This finding could be due to conceptual and methodological differences. While previous studies included reinforcement learning and reward expectancy in their definition of motivational control, we included measures of persistence on tasks.^16^ The lack of findings might also reflect our combination of nonverbal (at age 4.5) and verbal (at age 8.5) working memory tasks, which, although both loaded to a working memory construct, represented disparate skills at different developmental ages. In the same way, motivational control in our study was defined by delayed gratification at 3.5 years and persistence without the need for external reinforcement at 7 years, which might be measuring a different kind of motivational control despite exceptional model fit. It is important to note that we used a population-based sample with low to moderate levels of depressive symptoms overall, whereas previous studies demonstrating deficits in working memory and motivation consisted of individuals with clinical diagnoses of depression.^21,24^

Strengths of this study include a long prospective follow-up of mother–child dyads from pregnancy to 10 years of age, coupled with the use of multiple measures, including self-reports and objective, laboratory-based tasks. Our measures thus capture real-world manifestations of impairments that are not limited by reporter subjectivity. Furthermore, generating latent factors of EF domains allowed us to capture the multidimensionality of EF and reduced the impact of measurement error. However, this study has some limitations. Although a multitrait/multimethod approach allowed us to capture a holistic picture of EF across time and informants, it also has limitations. This was demonstrated in measures of hot EF, where laboratory-based tasks at age 7 and parent-reports showed low correlations. Low correlations among cross-informant items in latent variables have been previously reported, in particular, for observations of children across settings (laboratory-based vs mother-report) and for behaviors that are more challenging to observe (eg, internalizing and EF problems).^63,66^ Future research with larger sample sizes and consistent measurements should explore whether these associations persist across development and are bidirectional. In this study, we also found that Malay children reported higher levels of depressive symptoms compared with Chinese children. Given the multicultural makeup of Singapore and the high social coherence between ethnic groups, this finding may reflect Chinese participants underreporting depressive symptoms to prevent stigmatization or may be related to the general Chinese cultural norm of emotional restraint.^47^

The current study suggests clear associations between maternal depressive symptoms, impairments in EFs, and child depressive symptoms. However, the associations do not directly provide causal evidence. It is possible that deficits in EF are related to general psychopathology.^67^ Nevertheless, this study identifies EF as a potentially modifiable pathway and leverage point for prevention of depression in at-risk children. Long-term follow-up of randomized controlled trials of EF is required to understand whether EF interventions during childhood directly translate to improvements in emotional functioning.

Both hot and cold EFs explain variance in depression risk in school-aged children. Hot EF mediates the intergenerational transmission of depressive symptoms, whereas cold EF serves as a pathway linking maternal symptoms of depression to depressive symptoms in the child. Considering the pervasiveness of antenatal depressive symptoms, results can inform the development of EF-specific interventions that improve cognitive and emotional control in children from at-risk families.

## Supplementary Material

Supplementary Material

## Figures and Tables

**TABLE 1:** Demographic Characteristics of Study Samples

	GUSTO sample (N = 1,195)		Sample with child outcome (CDI) data (n = 739)		*p* ^a^
	n	(%)	n	(%)	
Sex, male	629/1,195	(52.7)	384/739	(52.0)	.77
Household income					
SGD < $2,000	167/1,097	(15.2)	105/690	(15.2)	1.00
SGD $2,000-$3,999	336/1,097	(30.6)	210/690	(30.4)	
SGD $4,000-$5,999	273/1,097	(24.9)	172/690	(24.9)	
SGD ≥ $6,000	321/1,097	(29.3)	203/690	(29.4)	
Maternal education level					
High school and below	355/1,161	(30.6)	210/730	(28.8)	.58
Diplomas/certificates	412/1,161	(36.3)	275/730	(37.7)	
University and above	394/1,161	(33.4)	245/730	(33.6)	
Ethnicity					
Chinese	666/1,192	(55.9)	428/737	(58.1)	.15
Indian	222/1,192	(18.6)	112/737	(15.2)	
Malay	304/1,192	(25.5)	197/737	(26.7)	
	**Mean**	**(SD)**	**Mean**	**(SD)**	
EPDS score	7.48	(4.47)	7.52	(4.41)	.58
Maternal age at delivery	31.23	(5.15)	31.35	(5.17)	0.69

**Note:** Sample size (mean). CDI = Child Depression Inventory; EPDS = Edinburgh Perinatal Depression Scale; GUSTO = Growing Up in Singapore Towards Healthy Outcomes.

a p *values were estimated from t test for continuous characteristics and from **χ**^2^ test for categorical characteristics*.

**TABLE 2:** Executive Function Measures and Association With Child Depressive Symptoms

Executive function^a^		*t*	β	(95% CI)	SE	*p*	η^2^_p_	Adjusted *p*^b^
Working memory	CANTAB task: between errors at 4.5 y	2.51	.13	(0.03, 0.23)	0.05	.013	0.02	**.036**
	CANTAB task: within errors at 4.5 y	2.43	.29	(0.05, 0.52)	0.12	.016	0.02	**.036**
	NEPSY word interference recall at 8.5 y	−2.27	−.40	(−0.75, −0.05)	0.18	.024	0.01	**.048**
	BRIEF working memory at 4.5 y	2.03	.13	(0.004, 0.27)	0.07	.043	0.01	.07
	BRIEF working memory at 7 y	3.50	.20	(0.09, 0.32)	0.06	.001	0.02	**.022**
Cognitive control	DCCS task: orientation accuracy % at 4.5 y	−2.53	−4.98	(−8.85, −1.11)	1.97	.012	0.03	**.036**
	DCCS orientation commission errors at 4.5 y	2.50	1.01	(0.21, 1.80)	0.40	.013	0.03	**.036**
	NEPSY switching at 8.5 y	−2.53	−.36	(−.64, −.08)	0.14	.012	0.02	**.036**
	Time perception accuracy at 4.5 y	−1.49	−.0007	(−.002, .0002)	0.0005	.137	—	.17
	BRIEF inhibitory control at 7 y	2.21	.14	(0.015, 0.26)	0.06	.028	0.01	.051
	BRIEF emotional control at 4.5 y	0.39	.03	(−0.11, 0.16)	0.39	.694	—	.69
	BRIEF emotional control at 7 y	2.42	.17	(0.32, 0.31)	0.07	.016	0.01	**.036**
Emotional control	Impossible puzzle anger and frustration at 7 y	0.86	.57	(−0.73, 1.86)	0.86	.391	—	.42
	DCCS task: emotion faces accuracy % at 4.5 y	−2.90	−10.93	(−18.37, −3.50)	3.77	.004	0.03	**.036**
	Emotion faces commission error at 4.5 y	2.78	2.35	(0.69, 4.01)	0.84	.006	0.03	**.036**
	Snack delay at 3.5 y	−1.17	−1.17	(−2.42, 0.08)	0.64	.066	—	.09
	Sticker delay at 3.5 y	−1.69	−.10	(−2.15, 0.16)	0.59	.091	—	.12
Motivational control	Time persisted in transparent box task at 3.5 y	−1.35	−.43	(−1.05, 0.19)	0.32	.177	—	.20
	Engagement in transparent box task at 3.5 y	−1.41	−1.38	(−3.30, 0.53)	0.98	.158	—	.19
	Impossible puzzle task persistence at 7 y	1.95	2.68	(−0.02, 5.39)	1.38	.052	—	.08

**Note:** BRIEF = Behavior Rating Inventory of Executive Function, Parent Rating Form; CANTAB = Cambridge Neuropsychological Test Automated Battery; DCCS = Dimensional Card Change Sort; NEPSY = Developmental Neuropsychological Assessment.

a Regression models included each individual measure with the following covariates: household income dichotomized (ie, SGD < $2,000 or ≥ $2,000), age at Child Depression Inventory assessment, sex of child, ethnicity of child, antenatal depression at 26 weeks, and child externalizing problems. Unstandardized **β** values are shown.

b p *values are adjusted for multiple comparisons using the false discovery rate method. Boldface indicates p values surviving multiple comparisons*.

## Data Availability

The datasets for this article are derived from the Growing Up in Singapore Towards Health Outcomes (GUSTO) longitudinal birth cohort study (https://gustodatavault.sg/). The data and materials necessary to reproduce the analyses presented here are publicly accessible. Data, study protocols, and data cleaning documentations can be shared by the GUSTO study team to achieve aims in a research proposal. Data access request for GUSTO datasets should be submitted in the GUSTO Form Application System (https://fas.sicsapps.com/site/login). The analytic code necessary to reproduce the analyses are available from the first author upon reasonable request. The analyses presented here were not preregistered.
