# Supplementary material for: Maternal Depressive Symptoms and Risk for Childhood Depression: Role of Executive Functions
Source: J Am Acad Child Adolesc Psychiatry. Author manuscript; Available in PMC 2026 Jul 6. (PMC13334413; doi:10.1016/j.jaac.2024.08.503)
Supplement: Supplementary Material [file NIHMS2184895-supplement-Supplementary_Material.docx]

**Supplemental Material**

**Figure S1.** Study flow diagram

**Figure S2.** Latent cognitive control as two separate constructs: a) cognitive flexibility and b) inhibitory control

**Figure S3.** Latent a) working memory and b) cognitive control constructs associated with child depressive symptoms

**Figure S4.** Latent a) emotional and b) motivational control constructs associated with child depressive symptoms

**Figure S5.** Latent growth curve model examining antenatal depression and the trajectory of maternal depression on child executive function

**Supplement 1.** Notes on sensitivity analysis for hot and cold EF model

**Table S1.** Task-based and reported measures of executive function variables

**Table S2.** Linear regression between ethnicity and depressive symptoms

**Table S3.** Linear regression between maternal and child depressive symptoms

**Table S4**. Correlation matrix of executive function tasks

**Table S5.** Factor loadings of measures on executive function latent variables

**Table S6.** Standardised coefficients of structural paths between executive function and their subdomains with antenatal depression and Child Depression Inventory (CDI)

**Table S7.** Sensitivity analysis: full model examining hot and cold EF using lab-based tasks

**Table S8.** Goodness of fit parameters for each executive function model

**Figure S1:** Study flow diagram

**
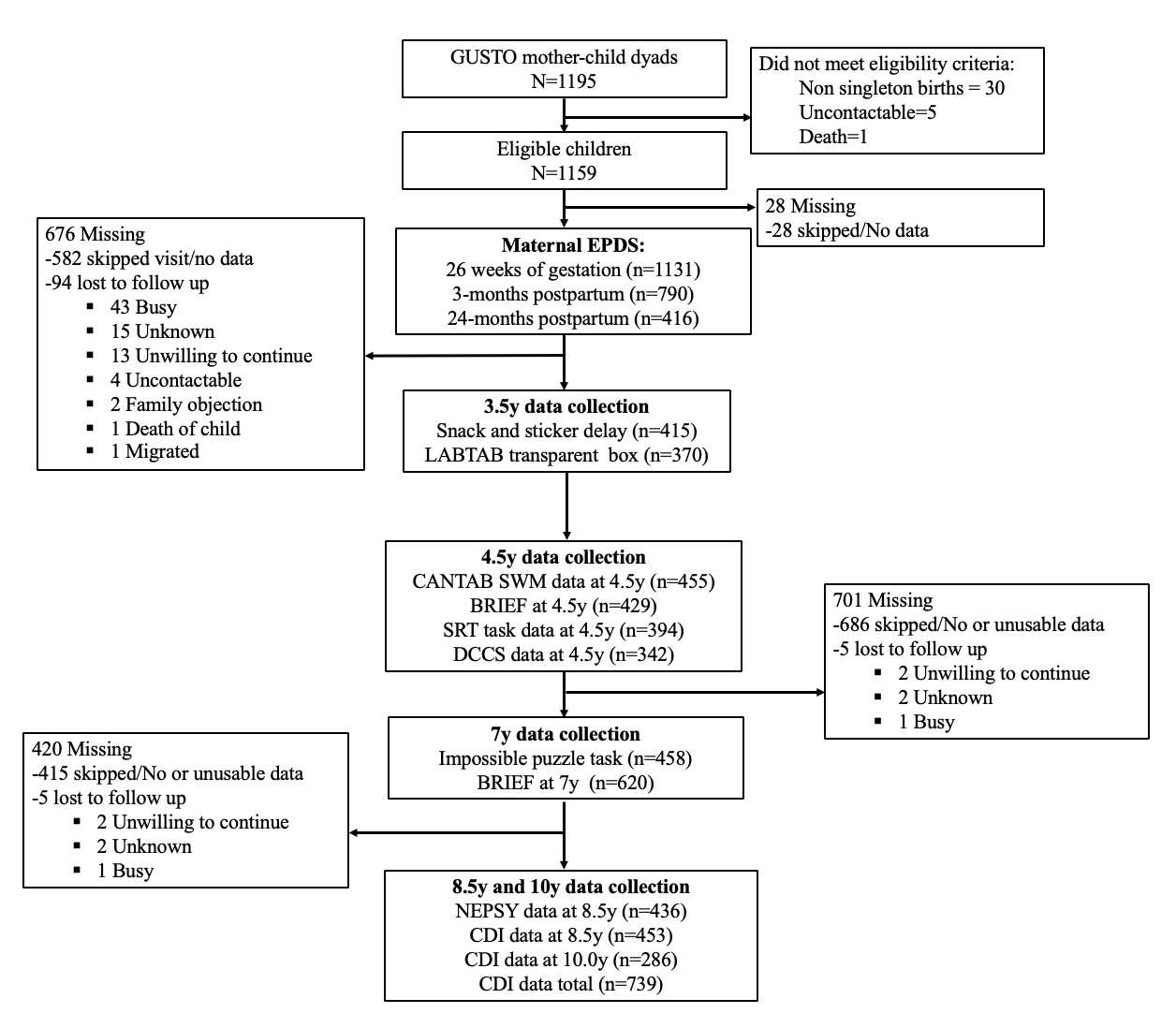
**

Note: GUSTO: Growing Up in Singapore Towards Healthy Outcomes; CDI: Child Depression Inventory; EPDS :Edinburgh Postnatal Depression Scale; DCCS: Dimensional Card Change Sort; BRIEF: Behavior Rating Inventory of Executive, Parent Rating Form; CANTAB: Cambridge Neuropsychological Test Automated Battery; LABTAB: Laboratory Temperament Assessment Battery; NEPSY: Developmental Neuropsychological Assessment (2^nd^ edition); SRT: Stop Reaction Time Task; CANTAB SWM: : Cambridge Neuropsychological Test Automated Battery: Spatial Working Memory;

**Figure S2:** Latent cognitive control as two separate constructs: a) cognitive flexibility and b) inhibitory control

**A. B.**


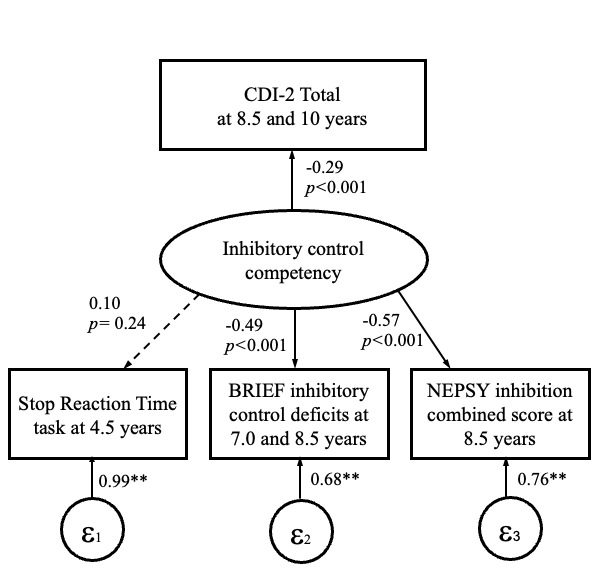

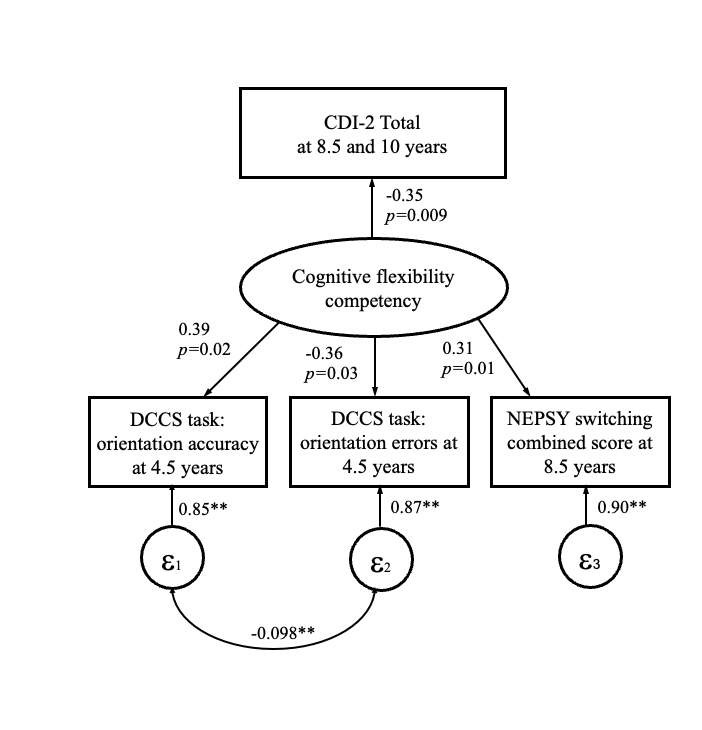


Note: CDI-II: Child Depression Inventory (2nd edition); DCCS: Dimensional Card Change Sort; NEPSY: Developmental Neuropsychological Assessment (2^nd^ edition); SRT: Stop Reaction Time task; CANTAB: Cambridge Neuropsychological Test Automated Battery

***p*< 0.001

**Figure S3**: Latent a) working memory and b) cognitive control constructs associated with child depressive symptoms^a^
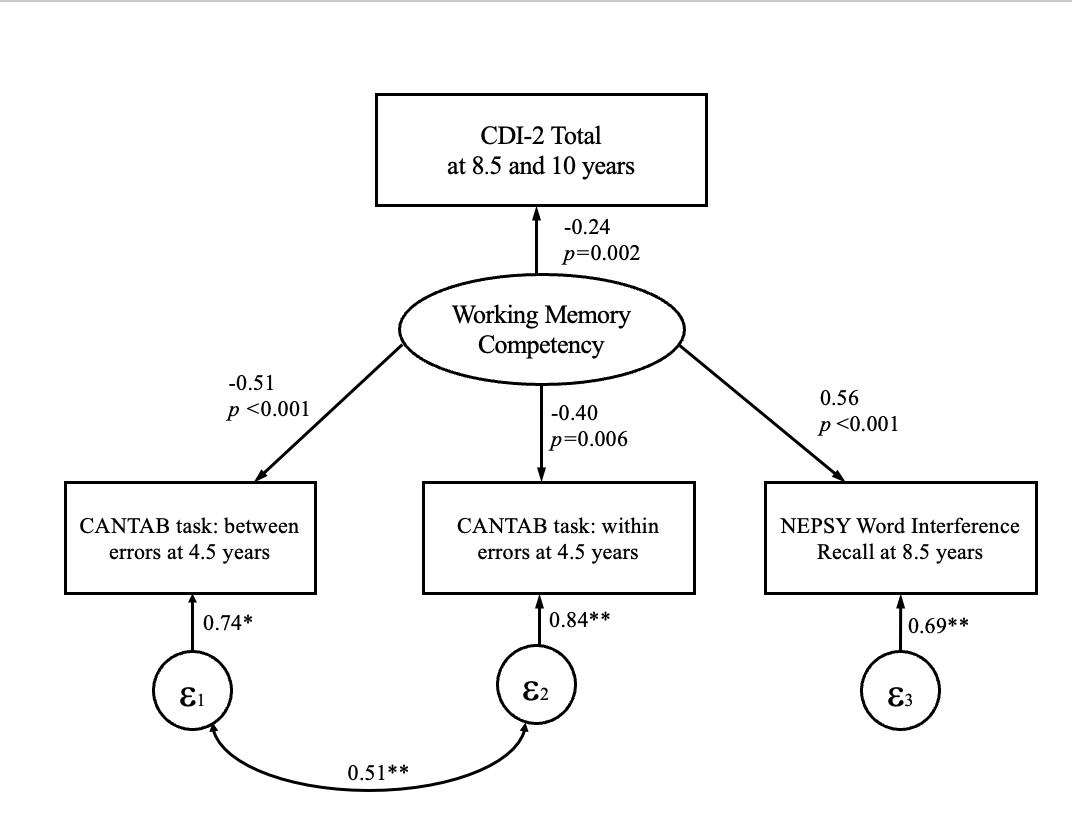


**A.**

**B.**


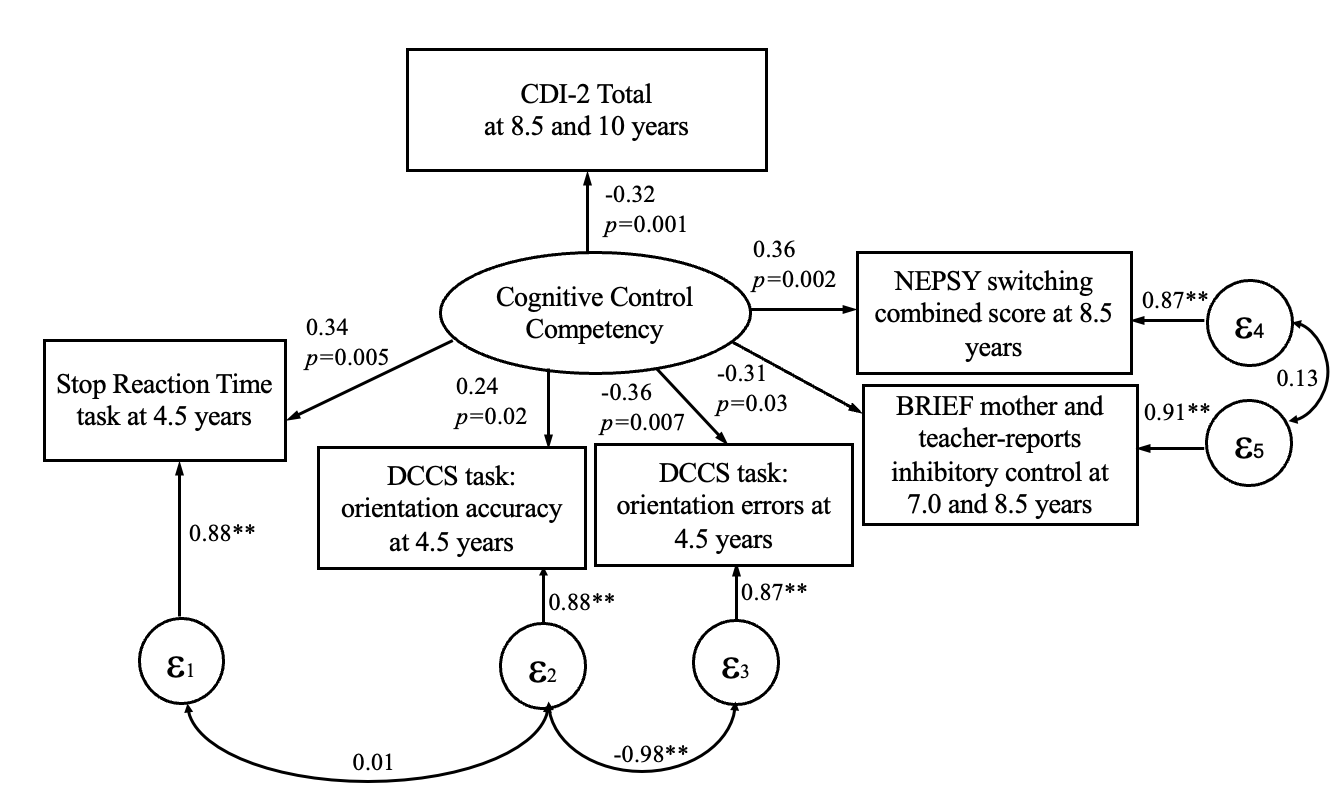


Note: CDI-II: Child Depression Inventory (2nd edition); DCCS: Dimensional Card Change Sort; NEPSY: Developmental Neuropsychological Assessment (2^nd^ edition); SRT: Stop Reaction Time task; CANTAB: Cambridge Neuropsychological Test Automated Battery. ^a^Correlation terms are added to measures collected within the same wave. ***p*< 0.001

**Figure S4:** Latent a) emotional and b) motivational control constructs associated with child depressive symptoms^a^

**
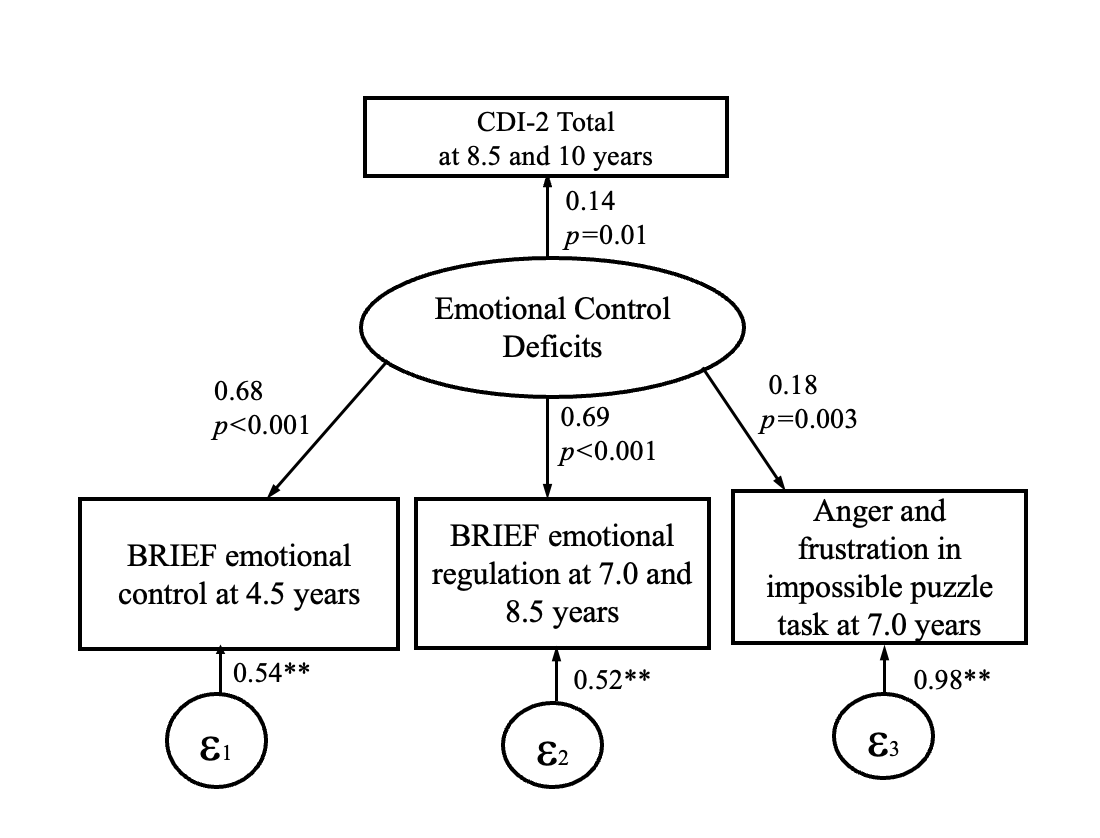
**

**A.**

**
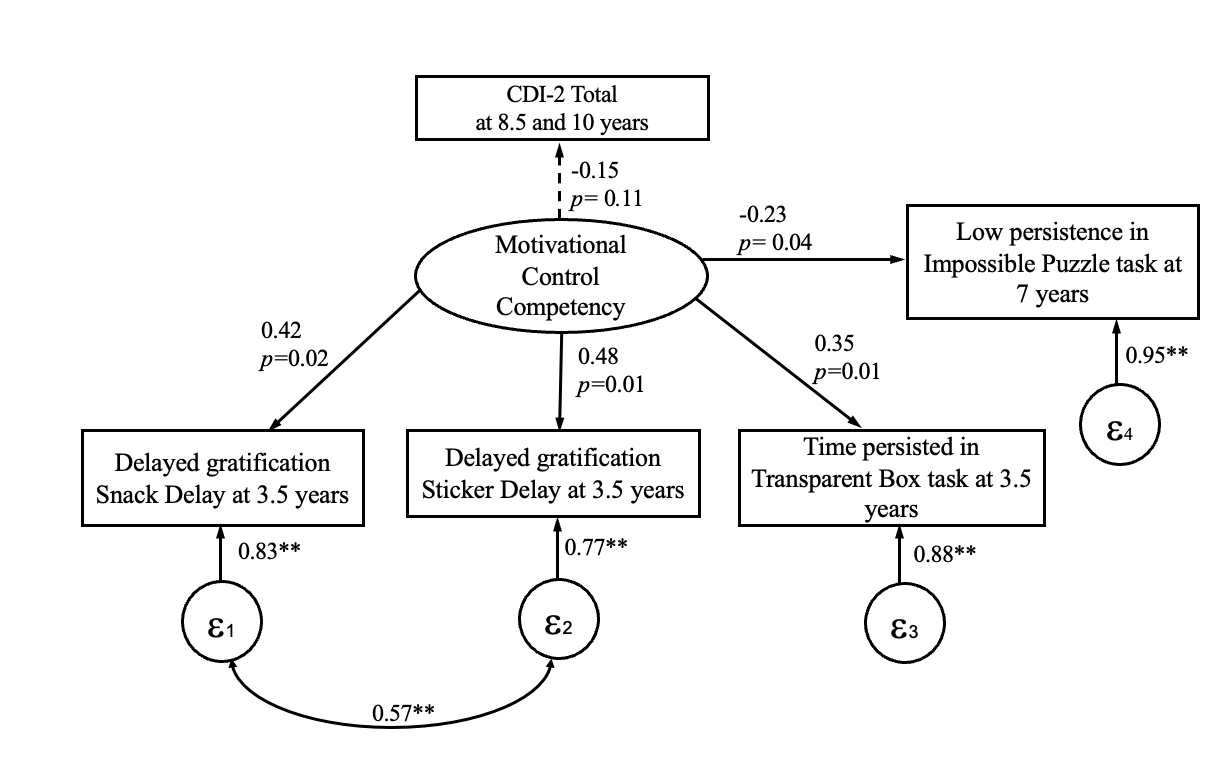
**

**B.**

Note: BRIEF: Behavior Rating Inventory of Executive, Parent Rating Form; CDI-II: Child Depression Inventory (2nd edition). ^a^ Correlation terms are added to measures collected within the same wave and same task. ***p*< 0.001

**Figure S5:** Latent growth curve model examining antenatal depression and the trajectory of maternal depression on child executive function and depression


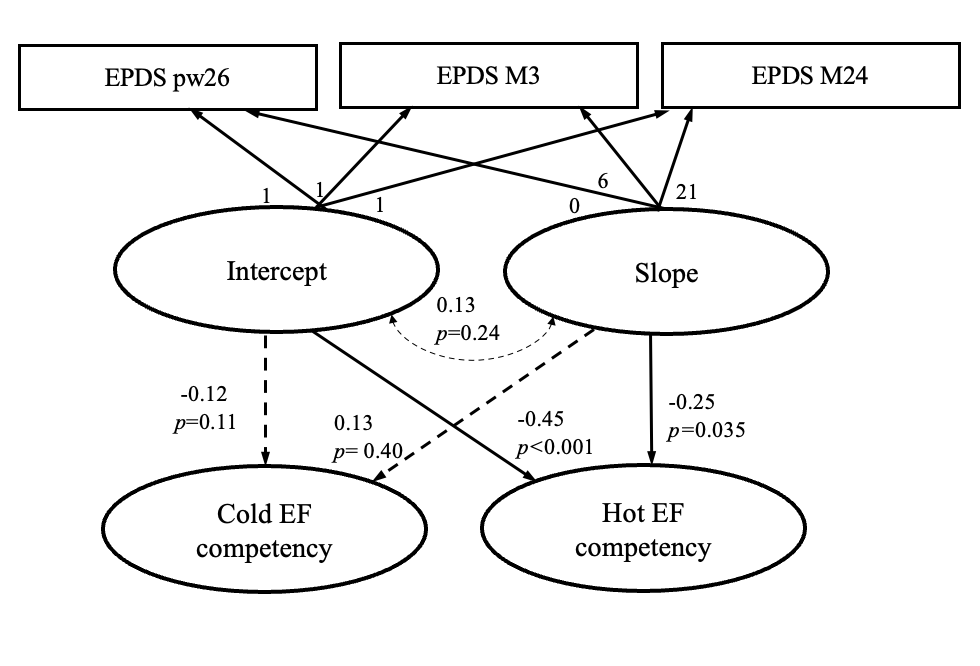


Note: EF: Executive Function; EPDS: Edinburgh Perinatal Depression Scale.

**Supplement 1:** Notes on sensitivity analysis for hot and cold EF model

A sensitivity analysis was performed to assess the whether the association found for hot EF can be explained by shared method variance, commonly found to inflate parent-child associations^1,2^. We first attempted to create a hot EF latent variable comprising of lab-based tasks from across ages 3.5 to 8.5 years. However, this construct demonstrated poor factor loadings, suggesting that EF lab-based tasks across these developmental stages may be measuring different constructs^2,3^. Given these results, we proceeded to split Hot EF lab-based tasks into early childhood (age 3.5 to 4.5) and late childhood (age 7). Compared to the early childhood hot EF latent variable; consisting of four different lab-based tasks, our analysis only consists of only one lab-based tasks at late childhood; the impossible puzzle (Table S8)

1. De Los Reyes A, Augenstein TM, Wang M, et al. The validity of the multi-informant approach to assessing child and adolescent mental health. Meta-Analysis. Psychol Bull. 2015;141(4):858-900. doi:10.1037/a0038498
2. Podsakoff PM, MacKenzie SB, Podsakoff NP. Sources of method bias in social science research and recommendations on how to control it. Annu Rev Psychol. Jan 10 2012;63:539-69. doi:10.1146/annurev-psych-120710-100452
3. Podsakoff PM, MacKenzie SB, Lee J-Y, Podsakoff NP. Common method biases in behavioral research: a critical review of the literature and recommended remedies. Journal of applied psychology. 2003;88(5):879.

**Table S1:** Task-based and reported measures of executive function variables

| **Latent Variable** | **Measure** | **Executive function Domain** | **Description** |
| --- | --- | --- | --- |
| Working Memory | Task: Cambridge Neuropsychological Test Automated Battery: Spatial Working Memory | Spatial working memory | Spatial working memory was assessed with the SWM from the Cambridge Neuropsychological Test Automated Battery (CANTAB). The child is tasked with searching for blue tokens through 6 coloured boxes presented on a computer screen. Each box contains only one token per trial. Searching a box more than once resulted in within errors and returning to a box that had already been emptied resulted in between errors. These indicate difficulties in sustaining mental representations. The fewer the between and within errors, the stronger the spatial working memory. |
|  | Task: Developmental Neuropsychological Assessment, Second Edition (NEPSY) Word List Interference | Verbal working memory | The child is aurally presented with 2 list of words in increasing lengths (2 to 5 words each). The child was first asked to repeat the two list in the same order (repetition trial). In the recall trial, the child was instructed to recall the first and second lists in the order of presentation. The total score out of 40 was converted into scaled scores (according to percentile ranks) and was used as an index of working memory. The higher the scaled score, the better the working memory. |
|  | Behaviour Rating Inventory of Executive Function-2nd Edition (BRIEF-2), parent- and teacher -reported | Working memory | General Working Memory was assessed from the working memory subscale in the BRIEF-2 questionnaire. The working memory subscale assesses the capacity to retain mental representations of information to achieve goals. There are 8 questions that the parent rates on the frequency of the behaviour/action. These items are rated on a frequency scale of 1-3 (1 = Never, 2 = Sometimes, 3 = Always) and summed. Converted T-scores were used, which accounted for the child’s age and sex. Lower working memory T-scores indicate better working memory performance in daily life. A composite score was created with parent and teacher reports. Parent reports were collected at age 7.0 years and teacher reports were collected at 8.5 years |
| Cognitive Control | Task: Dimensional Change Card Sort mixed trial | Cognitive flexibility | The paradigm is administered on a computer, using E-Prime version 2.0. Participants are asked to complete two paradigms. In the faces paradigm, four different targets cards were shown; a happy face and angry face in colours blue, green, orange and yellow. In the orientation paradigm, four target cards consisted of blue, green, orange and yellow emotionally neutral face in a right-side up or upside-down position. The child was provided with a similar set of test cards and was instructed to sort the test cards according to 1) emotion or colour in the faces paradigm and by 2) orientation or colour in the orientation paradigm and place them on the matching target cards. With every trial, the experimenter repeated the instructions. The first set of rules were to sort the test card according to the colour. After several trials, the child was were given a new rule to sort the test cards (i.e., by orientation). The accuracy and the total number of errors made (commission) following the new set of rules from both the faces and orientation paradigms were used as a measure of the child’s cognitive flexibility. The higher the accuracy, the higher the cognitive flexibility. |
|  | Task: Stop Reaction Time (RT) task | Inhibitory control | Time perception was assessed in the stop-RT task. The child was instructed by the experimenter to press the response pad as soon as a sequence of brief tones had ended 10ms in duration). There were six sequence lengths (comprising of 10 to 15 tones). Between each interval of tone sequences is the constant Stimulus Onset Asynchrony (SOA) and the child had to find the asynchronous sound to accomplish the objective. The child was given 10 practice trials before proceeding to testing. The child underwent three blocks of trials, with each trial a different duration of SOAs were used (250ms, 600ms and 1000ms). Each block contained 42 trials as the six sequence lengths were replicated 7 times for each length. The blocks were counterbalanced across participants to minimize order effects. Breaks were given between each block to reduce fatigue. An accuracy score for the 1000ms condition was used as a measure of time perception, with higher accuracy scores indicating better time perception derived from stronger inhibitory control. |
|  | Task: Developmental Neuropsychological Assessment, Second Edition (NEPSY) - Inhibition task | Cognitive flexibility | Black and white shapes and arrows are presented to the child and they are first asked to name either the type of shape (e.g., circle or square) or the direction of the arrow (e.g., up or down).The switching condition is when an opposite response is given depending on the direction of the arrow or shape presented (e.g., only provide an opposite response when you see an arrow facing upwards). The switching combined scaled score was used. This uses a scaled score metric integrates error rates and completion time with more weight given to accuracy than speed. A higher score indicates switching competency (i.e., cognitive flexibility). |
|  | Behaviour Rating Inventory of Executive Function-2nd Edition (BRIEF-2), parent- and teacher - reported | Inhibitory control | The Inhibit sub-scale measures the child’s ability to resist impulses. There is a total of 8 questions for this subscale. The parent rates the frequency of the behaviour/action on a scale of 1-3 (1 = Never, 2 = Sometimes, 3 = Always). These were summed and then converted into T-scores, which accounted for the child’s age and sex. Lower T-score indicates better inhibitory control. |
| Emotional control | Task: Impossible Puzzle | Anger and frustration | In the Impossible Tangram/Puzzle task, children were given pictures displaying 3 sets of tangrams (made from different triangles and squares in various orientations). They were then tasked to replicate these tangrams using the wooden shapes provided. The child was only allowed to work on one tangram at a given time and were given 5 minutes to solve tangram 1 and 2. 15-minutes were given for tangram 3 due to its difficulty. A trained researcher rated the child’s frequency of anger and frustration behaviours on all three trials on a Likert scale of 1 to 5. A high score indicates greater emotional control deficits. |
|  | Behaviour Rating Inventory of Executive Function-preschool and 2^nd^ edition (BRIEF-P), parent-reported | Emotional control and emotion regulation | The Emotional Control subscale was taken from the pre-school version of BRIEF. This measures the child’s ability to modulate or control their emotional expressions. The emotion regulation index from the 2nd edition of BRIEF comprises of two subscales: shifting and emotional control. This measures the ability to regulate emotional responses and adjust to changes in the environment. There is a total of 8 questions for each of these subscales, that the parent rates on the frequency of the behaviour or action. These items are rated on a frequency scale of 1-3 (1 = Never, 2 = Sometimes, 3 = Always) and summed. These were summed and then converted into T-scores, which accounted for the child’s age and sex. Lower T-scores indicate better emotional control and emotion regulation. |
| Motivational control | Task: Snack and sticker delay | Delay gratification | Delayed gratification was assessed with the sticker and snack delay task. The experiments were video-recorded and scored by one of three raters according to the protocol of Kochanska and colleagues. In the snack task, stimuli were colourful chocolates or cereals. In the sticker task, stimuli were colourful stickers with emoticons demonstrating different emotions. The child was first given a snack or a sticker in order to prime interest in the game. After eating the chocolate or pasting the sticker on the template, they were instructed to place both hands flat on a mat while the experimenter placed one chocolate or sticker under a transparent plastic cup positioned 5cm away from the top of the mat. The child was instructed to wait for the experimenter to ring a bell before retrieving the chocolate or sticker. The sequence in which the snack and sticker stimuli were presented to the child was matched for colour between versions. The procedure and scoring of the Sticker Delay task, with the exception of stimulus used, were identical with the Snack Delay task. The measure of effortful control is the child’s ability to resist reaching out for the stimulus. This behaviour was scored per trial and scores ranged from 1 to 7 and up to 2 additional points could be given if the child had maintained both hands on the mat throughout the task. The final score ranges from 1 to 9 with 9 indicating the highest inhibitory control. An average score across all four trials was calculated per task version and was used in all analyses. |
|  | Task: Laboratory Temperament Assessment Battery (LABTAB) -Transparent Box (Time spent) | Perseverance | In the Laboratory Temperament Assessment Battery (Lab-TAB) Transparent Box task, time spent is measured as the average amount of time (in seconds), the child engages with the task. A longer time spent engaging in the task, the greater the perseverance. |
|  | Task: Impossible puzzle | Perseverance | In the Impossible Tangram/Puzzle task, children were given pictures displaying 3 sets of tangrams (made from different triangles and squares in various orientations). They were then tasked to replicate these tangrams using wooden shapes provided. The child was only allowed to work on 1 tangram at a given time and was given 5 minutes to solve tangrams 1 and 2. 15-minutes was given for tangram 3 due to it being unsolvable. Whether the child continued working on the puzzle with the allocated time in the Tangram 3 (most difficult) condition or gave up was used as a measure of perseverance. This was coded as 1 = continued, 2 = gave up. A lower score indicates greater persistence and motivation. |

**Table S2:** Difference in depressive symptoms among ethnic groups

| **Ethnic group**  (Ref: Chinese) | **Maternal EDPS 26 weeks’ gestation** | | | | **Child CDI total problems** | | |
| --- | --- | --- | --- | --- | --- | --- | --- |
|  | ***β***  **(95% CI)** | **SE** | ***p*** | ***β***  **(95% CI)** | | **SE** | ***p*** |
| Malay | 0.22 | 0.07 | 0.002 | 0.27 | | 0.09 | 0.002 |
| Indian | 0.29 | 0.08 | <0.001 | -0.15 | | 0.11 | 0.17 |

Note: Standardized β values are shown. CDI: Child Depression Inventory (2^nd^ edition); EPDS: Edinburgh Perinatal Depression Scale.

**Table S3:** Linear regression between maternal and child depressive symptoms

| **Child depression subscales** | **EDPS 26 weeks’ gestation** | | | **EDPS 3-months postpartum** | | | **EDPS 24-months postpartum** | | |
| --- | --- | --- | --- | --- | --- | --- | --- | --- | --- |
|  | ***β***  **(95% CI)** | **SE** | ***p*** | ***β***  **(95% CI)** | **SE** | ***p*** | ***β***  **(95% CI)** | **SE** | ***p*** |
| CDI total problems | 0.22  (0.02, 0.42) | 0.10 | 0.035 | 0.02  (-0.20, 0.23) | 0.11 | 0.89 | 0.22  ( -0.05, 0.49) | 0.14 | 0.12 |

Note: CDI: Child Depression Inventory (2^nd^ edition); EPDS: Edinburgh Perinatal Depression Scale

**Table S4:** Correlation matrix of executive function tasks

|  | 1 | 2 | 3 | 4 | 5 | 6 | 7 | 8 | 9 | 10 | 11 | 12 | 13 | 14 | 15 | 16 | 17 | 18 | 19 | 20 |
| --- | --- | --- | --- | --- | --- | --- | --- | --- | --- | --- | --- | --- | --- | --- | --- | --- | --- | --- | --- | --- |
| 1. M41 Snack | 1.000 |  |  |  |  |  |  |  |  |  |  |  |  |  |  |  |  |  |  |  |
| 2. M41 Sticker | 0.611** | 1.000 |  |  |  |  |  |  |  |  |  |  |  |  |  |  |  |  |  |  |
| 3. M41_LabTab time persisted | 0.116* | 0.115* | 1.000 |  |  |  |  |  |  |  |  |  |  |  |  |  |  |  |  |  |
| 4. M41_LabTab engagement | 0.131* | 0.138* | 0.954** | 1.000 |  |  |  |  |  |  |  |  |  |  |  |  |  |  |  |  |
| 5. M54 SWM Between errors | -0.244** | -0.122* | -0.061 | -0.052 | 1.000 |  |  |  |  |  |  |  |  |  |  |  |  |  |  |  |
| 6. M54 SWM within errors | -0.248** | -0.111* | -0.084 | -0.086 | 0.608** | 1.000 |  |  |  |  |  |  |  |  |  |  |  |  |  |  |
| 7. M54 faces accuracy | 0.023 | 0.071 | 0.083 | 0.100 | -0.045 | -0.121* | 1.000 |  |  |  |  |  |  |  |  |  |  |  |  |  |
| 8. M54 faces errors | -0.040 | -0.108 | -0.098 | -0.112 | 0.022 | 0.128* | -0.959** | 1.000 |  |  |  |  |  |  |  |  |  |  |  |  |
| 9. M54 OR accuracy | 0.160** | 0.091 | 0.083 | 0.073 | -0.117* | -0.016 | 0.090 | -0.048 | 1.000 |  |  |  |  |  |  |  |  |  |  |  |
| 10. M54 OR errors | -0.175** | -0.091 | -0.071 | -0.065 | 0.115* | 0.024 | -0.071 | 0.042 | -0.978** | 1.000 |  |  |  |  |  |  |  |  |  |  |
| 11. M54 Stop reaction time | -0.025 | -0.029 | 0.022 | 0.039 | -0.158** | -0.166* | 0.114* | -0.132* | 0.080 | -0.075 | 1.000 |  |  |  |  |  |  |  |  |  |
| 12. Y7 impossible puzzle persist | -0.069 | -0.124 | -0.065 | -0.074 | 0.049 | 0.110 | -0.007 | 0.016 | -0.049 | 0.040 | -0.080 | 1.000 |  |  |  |  |  |  |  |  |
| 13. Y7 Impossible puzzle frustration | -0.030 | -0.046 | -0.075 | -0.035 | 0.096 | 0.032 | -0.080 | 0.057 | 0.015 | -0.028 | -0.063 | 0.101* | 1.000 |  |  |  |  |  |  |  |
| 14. Y8.5 NEPSY verbal recall | 0.239** | 0.201** | 0.009 | -0.005 | -0.279** | -0.201** | 0.134* | -0.108 | 0.103 | -0.108 | 0.133 | * -0.050 | -0.036 | 1.000 |  |  |  |  |  |  |
| 15. Y8.5 NEPSY switching | 0.166** | 0.139* | 0.019 | -0.010 | -0.270** | -0.224** | 0.112 | -0.099 | 0.119 | -0.109 | 0.120 | 0.015 | -0.013 | 0.252** | 1.000 |  |  |  |  |  |
| 16. M54 BRIEF emotional control | -0.108 | -0.08 | -0.044 | -0.058 | 0.054 | 0.077 | -0.051 | 0.075 | 0.130* | -0.103 | -0.009 | 0.096 | 0.139* | -0.016 | 0.050 | 1.000 |  |  |  |  |
| 17. M54 BRIEF working memory | -0.212** | -0.155** | -0.044 | -0.043 | 0.122* | 0.094 | -0.128* | 0.134* | 0.036 | -0.014 | -0.038 | 0.156** | 0.086 | -0.141* | -0.045 | 0.568** | 1.000 |  |  |  |
| 18. Y8.5 BRIEF working memory | -0.0937 | -0.1114 | -0.0560 | -0.0545 | -0.014 | 0.026 | -0.103 | 0.090 | -0.003 | 0.028 | -0.031 | 0.105* | 0.091 | -0.112* | -0.039 | 0.256** | 0.502** | 1.000 |  |  |
| 19. Y8.5 BRIEF inhibition | -0.0473 | -0.0741 | -0.1164 | -0.1082 | -0.0281 | 0.026 | -0.079 | 0.078 | -0.013 | 0.043 | -0.072 | 0.120* | 0.108* | -0.020 | 0.008 | 0.292** | 0.426** | 0.721** | 1.000 |  |
| 20. Y8.5 BRIEF emotion regulation | -0.0626 | -0.0990 | -0.1491* | -0.1459* | 0.0506 | 0.0693 | -0.1351* | 0.1512* | -0.020 | 0.051 | -0.075 | 0.077 | 0.141** | -0.021 | 0.005 | 0.455** | 0.413** | 0.668** | 0.766** | 1.000 |

**Table S5:** Factor loadings of measures on executive function latent variables

| **Factor** | **Factor loading** | ***SE*** | ***p-*value** |
| --- | --- | --- | --- |
| *Working Memory* | | | |
| Spatial working memory between errors at 4.5 years | -0.510 | 0.138 | <0.001 |
| Spatial working memory within errors at 4.5 years | -0.404 | 0.147 | 0.006 |
| NEPSY verbal working memory score at 8.5 years | 0.557 | 0.144 | <0.001 |
| *Cognitive Control* | | | |
| Stop reaction time inhibitory control at 4.5 years | 0.359 | 0.121 | 0.003 |
| Cognitive flexibility orientation accuracy at 4.5 years | 0.457 | 0.145 | 0.002 |
| Cognitive flexibility orientation errors at 4.5 years | -0.429 | 0.142 | 0.003 |
| NEPSY switching scaled score at 8.5 years | 0.350 | 0.121 | 0.004 |
| *Emotional Control* | | | |
| BRIEF emotional control at 4.5 years | 0.677 | 0.034 | 0.000 |
| BRIEF emotional regulation at 7.0 years | 0.693 | 0.040 | 0.000 |
| Anger and frustration in Impossible Puzzle at 7.0 years | 0.176 | 0.065 | 0.006 |
| *Motivational Control* | | | |
| Delayed gratification in Snack Delay at 3.5 years | 0.417 | 0.176 | 0.018 |
| Delayed gratification Sticker Delay at 3.5 years | 0.478 | 0.185 | 0.010 |
| Time Persisted in Transparent Box task at 3.5 years | 0.347 | 0.139 | 0.012 |
| Persistence in Impossible Puzzle task at 7.0 years | -0.225 | 0.111 | 0.043 |

Note: BRIEF: Behavior Rating Inventory of Executive, Parent Rating Form; NEPSY: Developmental Neuropsychological Assessment

**Table S6:** Standardised coefficients of structural paths between executive function and their subdomains with antenatal depression and CDI

| Structural paths | ***ß* (95% CI)** | | *p-*value |
| --- | --- | --- | --- |
| Subdomains of executive function on CDI | | | |
| Working memory | -0.24 (-0.40, -0.09) | | 0.002 |
| Cognitive control | -0.32 (-0.51, -0.14) | | 0.001 |
| Emotional control deficits | 0.14 (0.03, 0.26) | | 0.012 |
| Motivational control | -0.15 (-0.33, 0.03) | | 0.11 |
| Latent growth model | | | |
| Intercept on cold executive function | -0.11 (-0.25, 0.03) | 0.12 | |
| Slope on cold executive function | 0.12 (-0.18,0.42) | 0.44 | |
| Intercept on hot executive function | -0.45 (-0.59, -0.32) | <0.001 | |
| Slope on hot executive function | -0.26 (-0.49, -0.02) | 0.035 | |
| EPDS on CDI and executive functions | | | |
| EPDS on hot executive function | -0.26 (-0.38,-0.15) | | <0.001 |
| EPDS on cold executive function | -0.13 (-0.25,-0.004) | | 0.042 |
| Hot executive function on CDI | -0.14 (-0.25,-0.026) | | 0.016 |
| Cold executive function on CDI | -0.26 (-0.41,-0.11) | | 0.001 |
| EPDS on CDI | 0.006 (-0.08,0.09) | | 0.89 |
| *Mediation* | | | |
| Total effects from EPDS to CDI | 0.08 (0.003, 0.15) | | 0.042 |
| Total indirect effects from EPDS to CDI | 0.07 (0.02, 0.12) | | 0.006 |
| Indirect path from hot executive function | 0.03 (0.004, 0.07) | | 0.028 |
| Indirect path from cold executive function | 0.04 (-0.004, 0.07) | | 0.08 |
| Direct from EPDS to CDI | 0.006 (-0.08, 0.09) | | 0.89 |
| EPDS on subdomains of cold executive function | | | |
| EPDS on working memory | 0.07 (-0.02, 0.17) | | 0.14 |
| EPDS on cognitive control | -0.27 (-0.45, -0.08) | | 0.005 |
| Working memory on CDI | -0.08 (-0.49, 0.33) | | 0.70 |
| Cognitive control on CDI | -0.36 (-0.81, 0.09) | | 0.12 |
| EPDS on CDI | -0.01(-0.15, 0.13) | | 0.87 |
| *Mediation* | | | |
| Total effects from EPDS to CDI | 0.08 (0.005, 0.15) | | 0.037 |
| Total indirect effects from EPDS to CDI | 0.090 (-0.03, 0.21) | | 0.15 |
| Indirect path from cognitive control | 0.10 (-0.05, 0.22) | | 0.20 |
| Indirect path from working memory | -0.006 (-0.04, 0.03) | | 0.71 |
| Direct effects from EPDS to CDI | -0.012 (-0.15, 0.13) | | 0.87 |
| EPDS on subdomains of hot executive function | | | |
| EPDS on emotional control deficits | 0.27 (0.15, 0.36) | | <0.001 |
| EPDS on motivational control | 0.06 (-0.13,0.24) | | 0.56 |
| Emotional control deficits on CDI | 0.10 (-0.05,0.26) | | 0.18 |
| Motivational control on CDI | -0.10 (-0.32,0.13) | | 0.41 |
| EPDS on CDI | 0.06 (-0.04,0.14) | | 0.23 |
| *Mediation* | | | |
| Total effects from EPDS to CDI | 0.07 (-0.004, 0.14) | | 0.038 |
| Total indirect effects from EPDS to CDI | 0.02 (-0.03, 0.08) | | 0.40 |
| Indirect path from emotional control | 0.03 (-0.01, 0.07) | | 0.18 |
| Indirect path from motivational | -0.005 (-0.03, 0.016) | | 0.63 |

Note: CDI: Child Depression Inventory; EPDS: Edinburgh Perinatal Depression Scale

**Table S7:** Sensitivity analysis: full model examining hot and cold executive function using only lab-based tasks

| EPDS on CDI and executive functions (only lab-based tasks) | | |
| --- | --- | --- |
| Factor | Factor loading | *p* |
| *Cold EF* | | |
| NEPSY Verbal working memory score at 8.5 years | 0.43 | <0.001 |
| NEPSY switching scaled score at 8.5 years | 0.47 | <0.001 |
| Spatial working memory within errors at 4.5 years | -0.47 | <0.001 |
| Cognitive flexibility orientation condition at 4.5 years | 0.29 | 0.003 |
| Stop reaction time inhibitory control at 4.5 years | 0.34 | 0.004 |
| *Hot EF in early childhood* | | |
| Delayed gratification in Snack Delay at 3.5 years | 0.73 | 0.004 |
| Delayed gratification Sticker Delay at 3.5 years | 0.90 | <0.001 |
| Transparent box body anger at 3.5 years | -0.18 | 0.008 |
| Persistence on LABTAB box at 3.5 years | 0.20 | 0.001 |
| *Hot EF in late childhood* | | |
| Impossible puzzle anger and frustration at 7 years | 0.56 | 0.001 |
| Impossible puzzle enjoyment at 7 years | -0.19 | 0.013 |
| Impossible puzzle sadnesss at 7 years | 0.61 | <0.001 |
| Structural paths | *ß* (95% CI) | *p-*value |
| EPDS on hot early childhood EF | -0.04 (-0.15, 0.07) | 0.52 |
| EPDS on hot late childhood EF | -0.04 (-0.18, 0.10) | 0.58 |
| EPDS on cold EF | -0.13 (-0.25, 0.03) | 0.044 |
| Hot early childhood EF on CDI | -0.02 (-0.16,0.11) | 0.73 |
| Hot late childhood EF on CDI | 0.002 (-0.15, 0.16) | 0.98 |
| Cold EF on CDI | -0.26 (-0.41,-0.11) | 0.001 |
| CDI on EPDS | 0.04 (-0.04,0.12) | 0.28 |
| *Correlation between same-wave measures for Cold EF* | | |
| NEPSY Verbal working memory score and switching score at 8.5 years | 0.05 (-0.16 ,0.26) | 0.64 |
| Spatial working memory and cognitive flexibility at 4.5 years | 0.08 (-0.09, 0.25) | 0.37 |
| Spatial working memory and stop reaction time at 4.5 years | -0.09 (-0.27,0.09) | 0.36 |
| Cognitive flexibility and stop reaction time at 4.5 years | 0.14 (-0.08 ,0.35) | 0.22 |
| *Mediation* | | |
| Total effects from EPDS to CDI | 0.08 (0.004,0.15) | 0.038 |
| Total indirect effects from EPDS to CDI | 0.03 (-0.004,0.07) | 0.08 |
| Indirect path from cold EF | 0.03 (-0.005,0.07) | 0.09 |
| Indirect path from hot early childhood EF | 0.001 (-0.005,0.007) | 0.77 |
| Indirect path from hot late childhood EF | 0.00 (-0.006,0.006) | 0.98 |
| Direct path from EPDS to CDI | 0.04 (-0.04,0.12) | 0.28 |

**Table S8:** Goodness of fit parameters for each executive function model

| **Model** | **Number of free parameters** | ***χ^2^*** | ***AIC*** | ***RMSEA*** | ***CFI*** |
| --- | --- | --- | --- | --- | --- |
| Working memory on CDI | 13 | 0.49 | 5714.70 | <0.001 | 1.00 |
| Cognitive control on CDI | 21 | 12.51 | 9814.50 | 0.03 | 0.99 |
| Emotional control on CDI | 11 | 5.78 | 6298.79 | 0.03 | 0.96 |
| Motivational control on CDI | 16 | 2.28 | 6583.96 | <0.001 | 1.00 |
| LGCM: antenatal depression and its progression on executive function | 43 | 49.65 | 17378.99 | 0.007 | 1.00 |
| Hot and Cold executive functions on CDI | 38 | 42.57 | 12759.71 | 0.01 | 0.99 |
| Working memory and cognitive control on CDI | 36 | 52.76 | 10293.21 | 0.03 | 0.98 |
| Emotional control and motivational control on CDI | 31 | 19.400 | 10281.27 | <0.001 | 1.00 |
| *Sensitivity analysis: Hot and Cold EF using only lab-based tasks* | 49 | 100.71 | 15366.83 | 0.02 | 0.91 |

Note: CDI: Child Depression Inventory (2nd edition); LGCM: Latent growth curve model.
